# Mycorrhizal and Endophytic Fungi as a Tool for Sustainable Environments

**DOI:** 10.3390/plants14162581

**Published:** 2025-08-20

**Authors:** Raul S. Lavado, Viviana M. Chiocchio

**Affiliations:** Facultad de Agronomía, Universidad de Buenos Aires and Instituto de Investigaciones en Biociencias Agrícolas y Ambientales—INBA (CONICET/UBA), Av. San Martín 4453, Buenos Aires 1417, Argentina; chiocchi@agro.uba.ar

## 1. Introduction

The roots of vascular plants interact with different types of soil fungi, including arbuscular mycorrhizal fungi (AMF), dark septate endophytes (DSE), and other endophytes. These associations exhibit a wide range of symbiotic interactions [1]. The gradual accumulation of knowledge regarding these symbiotic processes enhances our understanding of ecological dynamics and the intricate connections among the various organisms inhabiting ecosystems worldwide. Furthermore, these associations can be leveraged as tools for maintaining sustainable environments [2,3].

The role of such symbioses is critical in natural environments, where they fulfill several essential functions under varying degrees of abiotic stress: they regulate the activities of plant pathogens and optimize nutrient uptake from both organic and inorganic sources. Endophytes produce precursors of plant hormones and other growth factors while also enhancing water absorption, thereby aiding plants in coping with water stress and mitigating the effects of salinity and other abiotic stresses [3,4].

Natural abiotic stresses often overlap with those induced by human activities, which directly and indirectly disturb the equilibrium among the components of ecosystems. Human activities have introduced various contaminants into the environment, including heavy metals, organic pollutants, plastics, pharmaceuticals, and petroleum derivatives [5,6,7,8,9,10]. Moreover, human activities are exacerbating the occurrence of abiotic stresses, such as soil salinity, acidity, and elevated air temperatures. Those stresses can negatively impact fungal populations and functions [11]. Despite this fact, the emerging functionalities of endophytes present promising avenues for restoring both agroecosystems and natural environments, contributing to their stability. In this context, fungi fulfill two critical roles:

i. Reducing the effects of inorganic pollutants on natural vegetation or crops, particularly heavy metals and salts [4];
ii. Degrading organic pollutants, such as hydrocarbons and agrochemicals, utilizing them as carbon sources [10].

## 2. Special Issue Contents

This Special Issue of *Plants* emphasizes the recent trend toward expanding the potential applications of microorganisms in agriculture and environmental management through their natural mechanisms. The aim is to address the ongoing and complex environmental crises related to biodiversity loss and other ecological challenges. The objective for this Special Issue is to showcase recent developments and future trends in the symbiotic interactions of fungi associated with cultivated or ruderal plants under various environmental conditions.

Cook et al. [12], studying apple rootstock genotypes, demonstrated the role of AMF in nutrient uptake and plant growth. They propose that aligning rootstock host genetics with compatible AMF species may enhance agricultural practices, particularly in fruit production during the nursery and orchard stages. In another study, Zhang et al. [13] identified fungal strains in the rhizosphere of buckwheat using morphological and molecular techniques, revealing significant enhancements in germination and plant growth. On the other hand, Yang et al. [14] found that certain strains exhibited phosphorus (P) solubilization effects that positively impacted the growth, root length, and yield of *Fagopyrum tataricum*, along with increases in the chlorophyll and soluble P content.

Xu et al. [15] investigated one legume and two grasses for rehabilitating degraded lands affected by various abiotic stresses. They found that AMF inoculation improved the plant uptake of nitrogen, P, and potassium (K), enhancing both aerial and root biomass. A significant correlation was observed between plant P and K contents and biomass. They identified the most suitable AMF for forage species aimed at restoring vegetation on degraded wastelands and proposed mechanisms for AMF–plant symbiosis that could bolster biomass yield.

While prior studies concentrated on endophyte effects on plant nutrition in crops or pastures, Catania et al. [16] explored the relationships between microorganisms in semi-arid forest environments. Although AMF typically form symbiotic relationships in most terrestrial plants, little evidence supports their infectivity in mosses, and there is a lack of studies on their nutritional impacts in this group. The authors identified AMF species and DSE predominantly in the stems and leaves contacting the soil, hypothesizing that fungal colonization aids in water and nutrient absorption. These findings contribute to understanding the ecological roles of these interactions and their importance in various ecosystems.

In light of the positive effects of plants growing in contaminated soils, Utge Perri et al. [17] examined the bioremediation potential of a specific AMF species for ecological restoration in post-mining landscapes. This area exhibited high concentrations of iron, lead, zinc, silver, and salts. The AMF species formed a symbiotic relationship with a pioneering perennial grass. In contrast, the AMF was also associated with ruderal species in non-contaminated soils, indicating that AMF display distinct ecological strategies in disturbed versus undisturbed environments.

Dark septate endophytes thrived in saline environments, where the forage *Chloris gayana* was introduced. Tobar Gómez et al. [18] studied the effect of salts on the fungus *Exserohilum rostratum*, isolated from this grass, and its contribution to the grass’ salinity tolerance. The fungus is able to tolerate salinity, but the significant effect of the fungus on the grass was recorded only in the salt-free treatments, and some effects were observed in the intermediate treatment, but the effect was null in the high-salinity treatment. The salt tolerance of this grass was not affected by its own fungus.

The impact of environmental stresses on plant–fungi interactions has primarily focused on the ability of microorganisms to mitigate the negative effects on plants or to degrade contaminants, as demonstrated in several recent studies and publications [4,19,20]. However, the effects of certain stresses on the growth and functionality of fungi remain inadequately understood. In this editorial, we aim to complement the findings presented in the Special Issue by briefly analyzing the effects of these stresses on fungi themselves.

Various natural and anthropogenic alterations to the environment—including soil nutrient enrichment or depletion and the presence of recalcitrant contaminants—disrupt higher plants and the ecological relationships between fungi and plants. Furthermore, endophyte behavior is influenced by numerous stresses, with notable differences in how fungi respond to each specific stress at varying concentrations and exposure durations, as well as differences between strains of the same fungal species [21,22].

One example is the use of fertilizers, which increases the biomass production of crops while affecting the contribution of endophytes to nutrient uptake [11]. Pesticides, designed to combat weeds, diseases, and insects, have been reported to exert varying effects on fungal populations, ranging from none or minimal to negative or even highly detrimental effects [23]. Endophytes are generally sensitive to high concentrations of heavy metals, which can delay, reduce, or even negate spore germination and the colonization potential of host plants. Endophytes exhibit differential responses to various salts; some fungi demonstrate negative effects from increased concentrations of chlorides and sulfates, while others show positive responses to carbonates [18]. Crude oil spills and their derivatives present significant environmental challenges globally, adversely affecting soil micro- and macro-organisms, a consequence that can persist for years. Certain fungal species thrive in petroleum-contaminated sites, metabolizing the contaminants as an energy source and mineralizing them to various extents [24]. Ureta Suelgaray et al. [25] identified variations in behavior among strains of the same fungi species following oil derivative contamination.

## 3. Additional Remarks

The effects of different types of stress are varied and complex. The effects on the physiology and growth of higher plants can be mitigated by the action of endophytic fungi. The topics considered in the present Special Issue address the importance of endophytic fungi in plant nutrition under different conditions and the influence of those microorganisms on the responses of plants, either crops or ruderal plants, to abiotic stresses. However, these positive effects on sustaining the environment can be affected by variations in fungal activity, due to the presence of such stresses.
